# Modifiable factors associated with a consistently high acute pain trajectory after surgical treatment of traumatic fractures in Ethiopia: a multi-center prospective cohort study

**DOI:** 10.1186/s13018-023-03770-0

**Published:** 2023-04-10

**Authors:** Mestawet Getachew, Anners Lerdal, Milada Cvancarova Småstuen, Million Tesfaye Eshete, Tilahun Desta, Maren Falch Lindberg

**Affiliations:** 1 Department of Clinical Pharmacy, School of Pharmacy, Institute of Health, Jimma University, Jimma, Ethiopia; 2 Department of Public Health Science, Faculty of Medicine, Institute of Health and Society, University of Oslo, Oslo, Norway; 3 Department of Interdisciplinary Health Sciences, Faculty of Medicine, Institute of Health and Society, University of Oslo, Oslo, Norway; 4 Department of Research and Administration, Lovisenberg Diaconal Hospital, Oslo, Norway; 5 Faculty of Health Science, Oslo Metropolitan University, Oslo, Norway; 6 Department of Orthopaedic Surgery, Lovisenberg Diaconal Hospital, Oslo, Norway; 7 Centre for Sustainable Healthcare Education, Faculty of Medicine, University of Oslo, Oslo, Norway; 8 Department of Orthopedics and Traumatology, St. Paul’s Hospital Millennium Medical College, Addis Ababa, Ethiopia

**Keywords:** Traumatic fracture, Growth mixture modeling, Acute postoperative pain, Trajectories, Risk factor

## Abstract

**Background:**

In Ethiopia, little is known about postoperative pain trajectories and possible predictive factors associated with them in patients undergoing surgery following traumatic fractures.

**Methods:**

This multi-center prospective observational cohort study included surgical candidates for traumatic fractures (*n* = 218). Worst pain intensity was measured with an 11-point numeric rating scale on the first 4 postoperative days and day of hospital discharge. Growth mixture modeling was used to identify subgroups of patients based on their pain trajectories, and logistic regression models to quantify associations between pain trajectories and demographic, clinical, psychological, and life style factors.

**Results:**

Two postoperative pain trajectory subgroups were identified: rapid pain relief (48% of included individuals) and consistently high pain (52% of included individuals). Sub-analysis stratified by cause of injury demonstrated that higher preoperative pain was an independent risk factor for consistently high postoperative pain regardless of the patient’s injury type: traffic accident (OR = 1.48, 95% CI 1.23–1.79), machine/tool injury or conflict (OR = 1.58, 95% CI 1.11–2.26), or fall (OR = 1.47, 95% CI 1.08–1.99). Moreover, longer surgical time was a risk factor for consistently high postoperative pain among patients who had a fall-related injury (OR = 1.02, 95% CI 1.00–1.03). In contrast, among patients with a traffic-related injury, receiving a nerve block was a protective factor (OR = 0.19, 95% CI 0.04–0.87) compared with general anesthesia.

**Conclusion:**

Higher preoperative pain and longer surgical time were associated with a consistently high acute postoperative pain trajectory. Clinicians may use these potentially modifiable factors to identify patients at risk for consistently high pain during the early postoperative period.

## Background

While several modalities are available for effective management of postoperative pain [1, 2], moderate or severe acute pain following orthopedic surgery is still a significant issue for a considerable proportion of surgical patients in both developed and developing countries [3–7]. In studies of patients undergoing a variety of surgical procedures in Ethiopia, 47–91% of patients experienced moderate to severe acute postoperative pain [8–13].

Previously, pain was expected to be proportional to the extent of tissue damage. However, the biopsychosocial model of pain proposes that patients’ pain experience is a result of extensive modulation of the pain signal through a complex interplay of biological, psychological and social factors, which are unique to each individual [14]. In a meta-analysis that mainly included studies from Western countries, the following preoperative predictors for poor acute postoperative pain control in multiple surgical specialties were identified: pain catastrophizing [15], younger age, female sex, smoking, presence of preoperative pain, history of anxiety and depressive symptoms, sleep difficulties, and higher body mass index [16]. Similarly, in studies of a variety of surgeries in developing countries including Ethiopia, previous surgery [13], lower educational status [11], younger age [11, 17], female sex [17], longer duration of surgery [9, 13], presence of preoperative pain and anxiety [13, 18], general anesthesia, peripheral nerve block [12, 18, 19] were identified as risk factors for moderate to severe pain.

Not surprisingly, the current state of knowledge on acute pain is based mainly on studies from western countries, and their findings cannot necessarily be generalized to populations in developing countries like Ethiopia with less available resources for pain management and different health systems, settings, patient education levels and lifestyles. Furthermore, among the studies conducted in developed and developing countries, different surgical procedures were included but there is a lack of studies within pain after traumatic fracture surgery. Thus, research on the extent of acute postoperative pain and associated risk factors among traumatic fractures surgical populations is of particular importance, primarily given the high frequency of traumatic fractures, in Ethiopia [20], as well as globally [21]. Besides, large variation exist in the severity of immediate postoperative pain i.e., two-fold higher in orthopedic surgery compared with laparotomy surgery [22]. Moreover, none of the above-mentioned studies evaluated pain trajectories. Assessment of intensity of pain at a single time point may be too simplistic because it does not take into account that resolution over time is a key feature of postoperative pain [23]. Evaluating patients’ acute pain trajectories over time may provide more clinically relevant information and a deeper understanding of patients’ individual differences [24]. Therefore, this study aimed to identify subgroups of patients with distinct acute pain trajectories, and determine the associations of pre and intraoperative factors with acute pain trajectory subgroups, in a cohort of Ethiopian patients who underwent surgery for traumatic fractures.

## Methods and materials

### Design and setting

This multi-center prospective observational cohort study included surgical candidates for traumatic fractures at two University teaching Hospitals in Ethiopia (i.e., Jimma Medical Center (JMC); Addis Ababa Burn Emergency and Trauma (AaBET) hospital). AeBET hospital is one of the largest government hospital in the country dedicated to emergency and trauma care, located in Addis Ababa, Ethiopia. Annually, this hospital has ~ 20,000 to 30,000 emergency visits. It is a 129-bed institution with a 52-bed orthopedic ward. The JMC is located in the city of Jimma in southwest Ethiopia. It provides services with a catchment population of over 20 million people. It has a total of 800 beds with 21 clinical service units. The surgery department has about 286 beds; including a 50-bed orthopedic ward.

### Sample size

We estimated that a sample of 200 patients would provide adequate power to estimate regression coefficients with sufficient precision with a model including 10 possible predictive factors. To allow for an estimated 10% attrition rate, we aimed to include a sample of 220 patients. With a final sample size of 220, our study had sufficient statistical power to evaluate these potentially predictive factors.

### Patient eligibility and recruitment

**Fig. 1:**
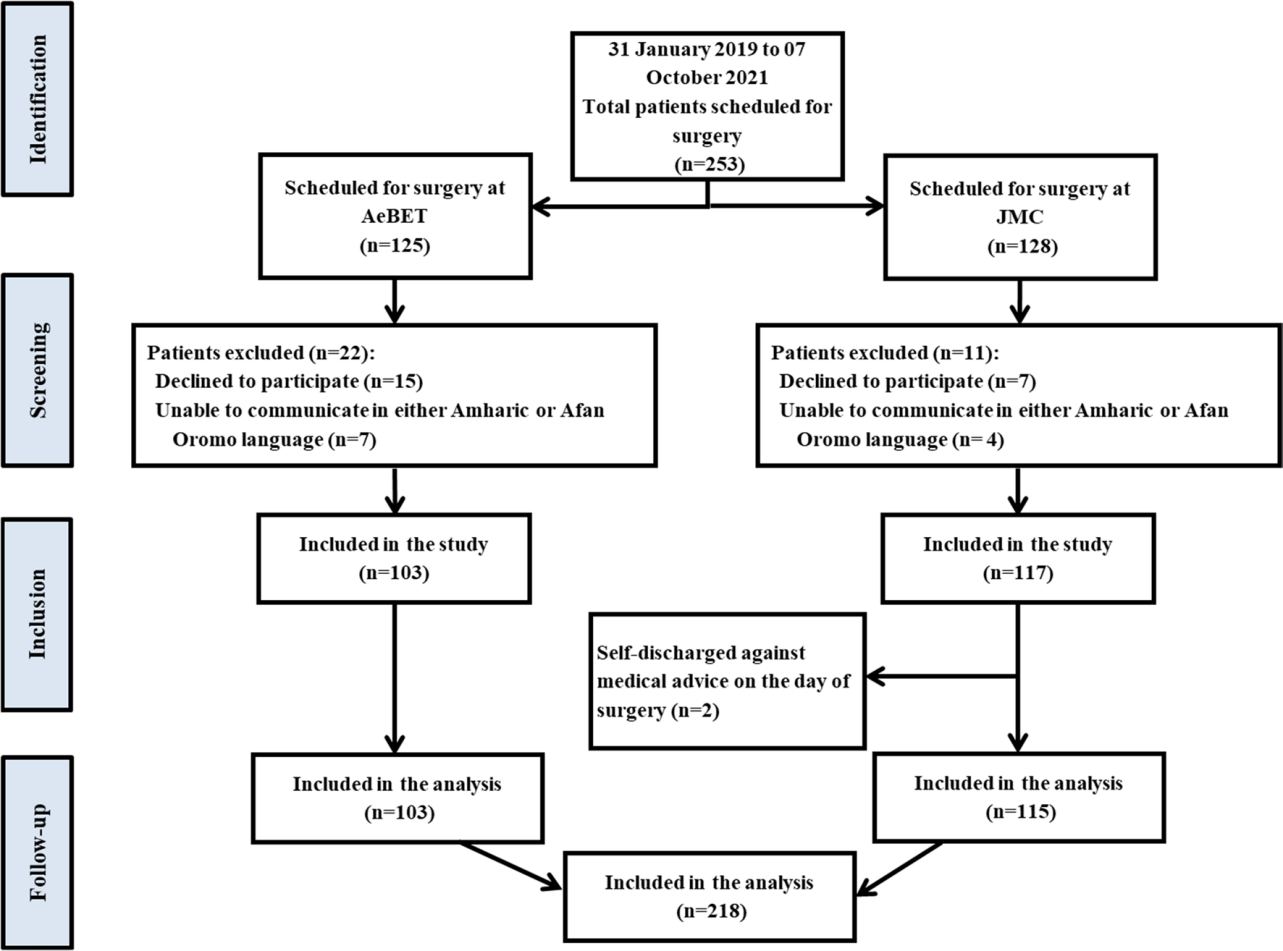
Flowchart of patient inclusion and exclusion

Patients scheduled to undergo elective surgery at one of the two study sites from 31 January 2019 through 07 October 2021 were consecutively approached by trained research assistants, who are bilingual speakers of Amharic and Afan Oromo. Patients were approached on the day before their planned surgery and were invited to participate if they met the following inclusion criteria: age 18 years or older, had an upper and/or lower extremity fracture related to injury, fully conscious with no cognitive impairment, and able to communicate in either Amharic or Afan Oromo language. As shown in Fig. 1, among the 253 patients screened for inclusion, 220 were considered eligible and a total of 218 were included in this analysis.

### Data collection procedures

Data were collected through patient chart review (i.e., age, sex, type of injury, location of injury, duration of surgery and, types of anesthesia) and by administering a questionnaire. The adult literacy rate of 51% reported in Ethiopia UNESCO Institute for Statistics, July 2017, many patients could not complete the questionnaire without help. Therefore, we used a face-to-face interviewer-administered questionnaire for all patients (i.e., the research assistant read the questions out loud and marked the patients’ responses in the questionnaire). The questionnaire was prepared in the two local languages (Amahric and Afan Oromo) that are predominantly spoken in the study’s settings.

On the day before surgery, the following preoperative information was obtained: patient age, sex, education, residence, life style variables (i.e., smoking, alcohol, khat), previous surgery, type of injury, and location of injury, as well as preoperative pain, anxiety, depression, and catastrophizing.

Intraoperative data, including duration of surgery and types of anesthesia, were collected from the patient’s medical chart on POD1.

Postoperatively, ratings of worst pain intensity at the surgical site over the last 24 h were collected on postoperative day (POD) 1, POD2, POD3, POD4, and the day of discharge. These pain ratings were the outcome and were used to determine patients’ acute postoperative pain trajectories.

### Measures

Anxiety and depression were assessed with the Hospital Anxiety and Depression Scale (HADS), a 14-item scale, scored on a 4-point Likert scale (0–3), ranging from “not at all” to “most of the time”. Seven items measuring anxiety and seven measuring depression over the last week yield a total score between 0 and 21 for each subscale [25]. The validated Ethiopian version of the HADS was used, with Cronbach’s alpha coefficients of 0.78, 0.76 and 0.87 for the anxiety and depression subscales and the full scale, respectively [26].

Patients’ level of pain catastrophizing was assessed with the Pain Catastrophizing Scale (PCS). The original PCS was developed by Sullivan et al. [27]. It contains 13 items with a 5-point Likert scale (0–4) ranging from “not at all” to “all the time”. The PCS yields a total score with a range of 0–52, with higher scores representing more catastrophizing. Since no Ethiopian version of the PCS existed at the time of the study, for the purpose of this study it was translated and adapted to Amharic and Afan Oromo, the two most widely spoken languages in the study settings. Translation of the original English version of the PCS was conducted according to a five-step process recommended for translating, adapting and validating instruments for cross cultural health care research [28].

Patients were asked to rate their level of pain at the surgical site over the last 24 h using the “worst pain” item of the Brief Pain Inventory short form (BPI), which has an 11-point numeric rating scale (NRS) with the end points 0 (no pain) and 10 (pain as bad as you can imagine). The BPI was originally developed as the Wisconsin Brief Pain Questionnaire to assess pain occurrence, intensity, interference with function, pain relief, and pain localization [29]. For this study, we used a validated Ethiopian version of the BPI [30].

### Statistical analyses

Categorical variables are reported as counts and percentages, and continuous variables as means and standard deviations. Differences between patients admitted at the two hospitals are assessed using *t-*tests for continuous variables and Chi-square tests for categorical variables.

The data were analyzed in two steps. In step 1, a growth mixture model (GMM) was fitted to identify subgroups of patients based on similarities in their trajectories of acute postoperative pain across five time points (POD1-4, day of discharge). We identified two distinct trajectories, namely the rapid postoperative pain relief class and the consistently high postoperative pain class. In step 2, the two classes were used as the dependent variable and we fitted a multiple logistic regression model to identify possible associations with pre- and intra-operative factors (i.e., those that have been previously shown to influence acute postoperative pain). Further, we stratified our data by cause of injury and fitted separate logistic regression models. The results are presented as odds ratios (OR) with 95% confidence intervals (CI) and *p* values was considered significant when *p* < 0.05. To increase precision of the estimates, we used bias-corrected bootstrapping with 1000 samples. All analyses were considered exploratory, so no correction for multiple testing was performed. Data analyses were performed using the Statistical Package for Social Science (SPSS) version 26 and Stata version 17.

## Results

### Study population

Initially, 220 patients were included and completed the baseline data. Two patients were excluded after self-discharging against medical advice on the day of surgery. Postoperative pain scores were assessed for 218 patients with a retention rate of 99.1% at POD1. Of these, 99.1%, 96.3%, 88.1%, and 99.1% also had pain ratings for POD2, POD3, POD4 and day of discharge, respectively.

### Patient characteristics

The demographic, lifestyle, clinical and psychological characteristics of the patients at each hospital are summarized in Table 1. Several preoperative differences were found between patients admitted to the two hospitals (i.e., history of alcohol and khat use, duration of surgery, preoperative pain intensity, anxiety, depression).

**Table 1:** Patient characteristics by hospital

Characteristic	Patients at JMC hospital *n* = 115	Patients at AaBET hospital *n* = 103	P value
	mean (SD)	mean (SD)	
Age (years)	33.3 (11.6)	33.5 (11.7)	0.89
	*n* (%)	*n* (%)	
Sex (Male)	92 (80)	84 (81.6)	0.77
*Education status*			
Primary	43 (46.2)	45 (49.5)	0.90
Secondary	34 (36.6)	31 (34.1)	
Diploma and higher	16 (17.2)	15 (16.5)	
*Residence*			
Urban	63 (54.8)	49 (47.6)	0.29
Rural	52 (45.2)	54 (52.4)	
*Cause of injury*			
Traffic accident	56 (48.7)	58 (56.9)	0.22
Machine/tool injury or conflict	33 (28.7)	19 (18.6)	
Fall	26 (22.6)	25 (24.5)	
*Location of injury*			
Upper extremities	52 (45.2)	34 (33.0)	0.18
Lower extremities	58 (50.4)	64 (62.1)	
Both upper and lower extremities	5 (4.3)	5 (4.9)	
*Lifestyle variables*			
Alcohol (yes)	63 (54.8)	39 (37.9)	0.012
Khat (yes)	60 (52.2)	34 (33.0)	0.004
Smoking (yes)	16 (13.9)	9 (8.7)	0.23
*Anesthesia type*			
General	41 (35.7)	38 (38.0)	0.47
Spinal	48 (41.7)	46 (46.0)	
Nerve block	26 (22.6)	16 (16.0)	
*Clinical variables*			
Previous surgery (Yes)	16 (13.9)	8 (7.8)	0.15
Consistently high postoperative pain (Yes)	26 (22.6)	87 (84.5)	< 0.001
	mean (SD)	mean (SD)	
Preoperative worst pain intensity (range 0–10)	4.2 (2.5)	7.1 (2.5)	< 0.001
Duration of surgery (minutes)	99.2 (46.6)	147.6 (58.8)	< 0.001
*Psychological variables*			
Anxiety (range 0–21)	7.5 (4.1)	9.6 (4.3)	< 0.001
Depression (range 0–21)	13.1 (3.2)	10.8 (3.5)	< 0.001
Pain catastrophizing (range 0–52)	25.8 (9.1)	25.8 (7.9)	0.99

JMC = Jimma Medical Center; AaBET = Addis Ababa Burn Emergency and Trauma

*P-values* < *.05* were considered statistically significant

### Acute postoperative worst pain intensity trajectories

**Fig. 2:**
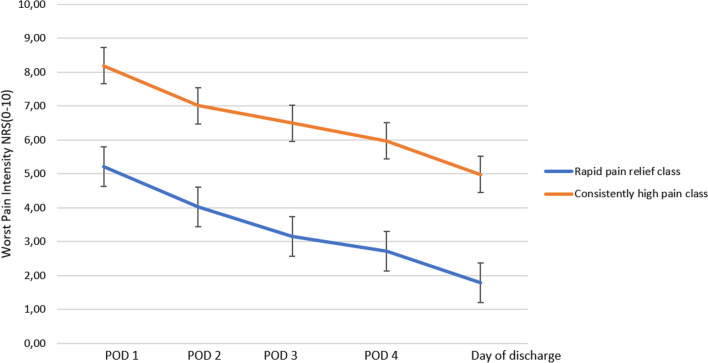
Mean postoperative pain intensity score and 95% confidence intervals for 2 trajectory classes: rapid postoperative pain relief (*n* = 105) and consistently high postoperative pain (*n* = 113)

The GMM model based on patients’ pain ratings at five time points during the acute postoperative period identified two classes of patients with distinct pain trajectories. As shown in Fig. 2, patients in class 1 (*n* = 105, 48.2%) experienced moderate pain intensity (mean = 5.2, 95% CI 4.9–5.5) on POD1, followed by a rapid decline. Patients in class 2 (*n* = 113, 51.8%) experienced severe pain intensity (mean = 8.2, 95% CI 8.0–8.4) on POD1, followed by consistently high pain intensity over the five postoperative time points. To reflect the pattern of the trajectories, we labeled class 1, “rapid postoperative pain relief" and labeled class 2 “consistently high postoperative pain”.

### Factors associated with consistently high postoperative pain

Possible predictive factors for membership in the consistently high postoperative pain class are listed in Table 2. Of the sociodemographic variables, neither age, sex, type of residence nor level of education were significantly associated with type of pain trajectory. However, those who suffered machine/tool-related injuries or were involved in conflict were almost 80% less likely to be in the consistently high postoperative pain class compared to those with traffic-related injuries (OR = 0.19, 95% CI 0.06–0.63).

**Table 2:** Multivariable logistic regression of factors associated with consistently high postoperative pain class membership

Variables	Full		
	OR	95% CI	*P value*
*Socio-demographic*			
Age, years	1.03	0.99–1.07	0.17
Female sex (Ref: male)	0.48	0.13–1.78	0.27
*Education status*			
Secondary (Ref: primary)	2.10	0.34–12.80	0.42
Higher (Ref: primary)	0.17	0.02–1.44	0.16
*Residence*			
Rural (Ref: urban)	3.42	0.61–19.30	0.16
*Cause of injury*			
Machine/tool injury or conflict (Ref: traffic accident)	0.19	0.06–0.63	0.006
Fall (Ref: traffic accident)	0.32	0.02–1.22	0.07
*Lifestyle*			
Khat (Ref: never used)	0.38	0.14–1.02	0.054
Smoking (Ref: never smoked)	2.70	0.68–10.74	0.16
Alcohol (Ref: never used)	0.36	0.14–0.91	0.03
*Clinical*			
Preoperative worst pain intensity (range: 0–10)	1.38	1.16–1.64	< 0.001
Duration of surgery (in minutes)	1.01	1.00–1.02	0.02
Previous surgery (Ref: No previous surgery)	1.34	0.31–5.85	0.69
*Anesthesia type*			
Spinal (Ref: general anesthesia)	0.44	0.16–1.19	0.10
Nerve block (Ref: general anesthesia)	0.21	0.05–0.79	0.02
*Psychological*			
Anxiety (range 0–21)	1.06	0.94–1.19	0.38
Depression (range 0–21)	0.80	0.69–0.93	0.003
Pain catastrophizing (range 0–52)	1.04	0.98–1.12	0.19

Rapid postoperative pain relief class as reference

OR = odds ratio; CI = confidence interval

*P-values* < *.05* were considered statistically significant, Ref = reference

With respect to life style, use of khat was associated with reduced likelihood of belonging to the consistently high postoperative pain class; however, this association did not reach the level of statistical significance. Moreover, alcohol users were about 70% less likely to be in the consistently high postoperative pain class compared to non-users.

Regarding clinical characteristics, preoperative worst pain intensity was significantly associated with being in the consistently high postoperative pain class. For each unit increase on the preoperative pain intensity scale, the odds of being in the consistently high postoperative pain class increased by 38% (OR = 1.38, 95% CI 1.16–1.64). Duration of surgery was significantly associated with higher odds for being in the consistently high postoperative pain. Furthermore, for type of anesthesia, patients with nerve block anesthesia had about 80% lower odds than those who had general anesthesia of being in the consistently high postoperative pain class (OR = 0.21, 95% CI 0.05–0.79). Of the psychological factors, only depression remained independently associated with type of pain trajectory. The higher the depression score, the lower the odds of being in the consistently high postoperative pain class (OR = 0.80, 95% CI 0.69–0.93).

### Factors associated with consistently high postoperative pain by cause of injury

Predictive factors for membership in the consistently high postoperative pain class stratified by cause of injury are shown in Table 3. Preoperative worst pain intensity remained an independent predictive factor for being in the consistently high postoperative pain class for all causes of injury. A 1-point increase in preoperative worst pain intensity was associated with about a 50% increase in the odds of being in the consistently high postoperative pain class.

**Table 3:** Multivariable logistic regression models of factors associated with consistently high postoperative pain class membership^§^ by cause of injury

Variables	Traffic-related injury (*n* = 108)			Machine/tool-related injury or conflict (*n* = 52)			Fall-related injury (*n* = 50)		
	OR	95% CI	*P value*	OR	95% CI	*P value*	OR	95% CI	*P value*
*Lifestyle*									
Alcohol (Ref: never used)	0.65	0.25–1.65	0.36	0.43	0.08–2.45	0.34	0.30	0.06–1.58	0.16
*Clinical characteristics*									
Preoperative worst pain intensity (range: 0–10)	1.48	1.23–1.79	< 0.001	1.58	1.11–2.26	0.01	1.47	1.08–1.99	0.01
Duration of surgery (minutes)	1.00	0.99–1.01	0.26	1.01	0.99–1.04	0.08	1.02	1.00–1.03	0.04
*Types of anesthesia*									
Spinal (Ref: general)	0.58	0.21–1.65	0.31	0.34	0.05–2.34	0.27	1.30	0.23–7.38	0.76
Nerve block (Ref: general)	0.19	0.04–0.87	0.03	0.12	0.01–1.56	0.10	0.47	0.05–4.29	0.51
*Psychological*									
Depression (range: 0–21)	0.88	0.77–1.02	0.08	0.84	0.64–1.12	0.24	0.96	0.77–1.19	0.70

^**§**^Rapid postoperative pain relief class as reference; Abbreviations: OR = odds ratio; CI = confidence interval

*P-values* < *.05* were considered statistically significant; Ref = reference

Type of anesthesia (i.e., nerve block vs general anesthesia) remained associated with the consistently high postoperative pain class only for those with a traffic-related injury (OR = 0.19, 95% CI 0.04–0.87).

Duration of surgery was significantly associated with the consistently high postoperative pain class only for those who had fall-related injuries (OR = 1.02, 95% CI 1.00–1.03). For each 10 min increase in the duration of the surgery the odds of being in the consistently high postoperative pain class increased by 22%.

## Discussion

To our knowledge, this is the first multi-center study on acute pain trajectories in a cohort of Ethiopian patients who underwent surgery for traumatic fractures. We identified two subgroups of patients with distinct trajectories of acute pain: the “rapid postoperative pain relief class” and the “consistently high postoperative pain class” based on five daily pain ratings during the early postoperative period.

More than half of the patients in this study were classified as having consistently high postoperative pain, which suggests that acute postoperative pain is a major problem for patients with traumatic fractures in Ethiopia. Postoperative pain management may be a particular challenge in a developing country. Several barriers to postoperative pain management have been identified in previous studies in Ethiopia [31, 32], such as lack of health personnel trained in pain management, lack of access to analgesics and adjuvant analgesics, lack of pain treatment protocols, fear of patient addiction to pain-relieving drugs, misjudgment of patients’ pain severity, and strict drug regulations.

Patients who had an injury caused by machine/tool use or conflict had an 80% decrease in the odds of having a consistently high postoperative pain trajectory, compared to those with injuries caused by a traffic accident. According to Hu et al. [33], patients experience extensive bodily pain beginning in the immediate upshot of motor vehicle collisions, which does not remit, but can instead go on to a gradual extension of pain from localized to widespread over time, suggesting that these patients may also be at risk for developing persistent pain conditions. Thus, the non-improvement of pain in patients who had a traffic-related injury may suggest that they had more complex injuries, leading to more severe acute pain that may warrant a multimodal and more invasive approach to pain management.

The subgroup analysis revealed that for patients with traffic-related injuries, receiving nerve block anesthesia was protective against a consistently high postoperative pain trajectory, compared to general anesthesia. This is likely due to the advantage of nerve block anesthesia for postoperative pain management. Several other studies done in Ethiopia [12, 18], Tanzania [19], Brazil [34], and France [35] reported the same phenomenon, indicating that patients who had general anesthesia had a higher intensity of postoperative pain when compared to regional anesthesia. Moreover, a clinical practice guideline strongly acknowledged clinicians to consider local or regional block anesthesia during operative fixation of fractures and as part of postoperative multimodal pain management [36]. Thus, clinicians need to pay attention to those with traffic-related injuries.

The sub-analysis regression model stratified by cause of injury is unique to this study. Our main finding is that higher preoperative pain was a stable risk factor for having consistently high postoperative pain across all injury types. This finding is consistent with a recent systematic review and meta-analysis, which identified that the presence of preoperative pain is a known predictor for poor acute postoperative pain control [16]. Furthermore, among patients who had a fall-related injury, longer duration of surgery was significantly associated with consistently high postoperative pain in our study. This finding is in accordance with previous published research [9, 13]. Prolonged surgical duration is associated with greater surgical stress to the body and likely greater tissue trauma. Thus, clinicians working in trauma centers can use this information to better assess which patients may require additional pain control.

This study has several strengths and limitations that need to be considered. The strengths of this study are: prospective multi-center design, the low attrition rate, a relatively representative population that included patients from one of the largest trauma centers and one teaching and referral hospital in Ethiopia, a relatively large sample size and the advanced statistical analysis. Our study has some limitations that should be considered when interpreting the results. The results only apply to patients undergoing surgery for traumatic fractures and do not control for potentially confounding factors such as perioperative administration of analgesia. In addition, several differences were found between the two hospitals with respect to lifestyle-related (history of alcohol and khat use), clinical (preoperative pain, length of surgery) and psychological (anxiety, depression) factors. The patients admitted to the two hospitals live in different regions of the multi-ethnic Ethiopia, which may result in cultural, contextual, religious, and psychological differences in the patient populations for the two hospitals. While these differences resulted in a more heterogeneous sample, our decision to include patients from two hospitals in two different regions of Ethiopia may have resulted in more generalizable findings.

## Conclusions

The present study shows two classes of patients based on their acute postoperative pain trajectories, and more than half of patients were classified in the consistently high pain trajectory class. Higher levels of preoperative worst pain intensity and longer surgical duration were significant factors related to the consistently high acute postoperative pain trajectory. Clinicians working at trauma centers should be aware of these factors to identify patients at risk of the consistently high acute postoperative pain trajectory and take measures to minimize early postoperative pain. Nerve block anesthesia was associated with lower risk of a more severe pain trajectory in patients with traffic-related injuries. Clinicians need to be aware of patients with traffic-related injuries scheduled for operative fixation of fractures and consider using a multimodal pain management regiment in line with current guidelines.

## Data Availability

The datasets used and/or analyzed on this study are available from the first author (Mestawet Getachew) on reasonable request.

## Abbreviations

JMC: Jimma Medical Center
AaBET: Addis Ababa Burn Emergency and Trauma
UNESCO: United Nations Educational, Scientific and Cultural Organization
GMM: Growth Mixture Model
POD: Postoperative day
HADS: Hospital Anxiety and Depression Scale
PCS: Pain Catastrophizing Scale
BPI: Brief Pain Inventory short form
NRS: Numeric Rating sSale
